# LL37-mtDNA regulates viability, apoptosis, inflammation, and autophagy in lipopolysaccharide-treated RLE-6TN cells by targeting Hsp90aa1

**DOI:** 10.1515/biol-2022-0943

**Published:** 2024-08-28

**Authors:** Yunlong Zuo, Run Dang, Hongyan Peng, Peidan Hu, Yiyu Yang

**Affiliations:** Pediatric Intensive Care Unit, Guangzhou Women and Children’s Medical Center, Guangzhou Medical University, No. 318, Renmin Middle Road, Yuexiu District, Guangzhou, Guangdong, 510120, China

**Keywords:** LL37, mtDNA, Hsp90aa1, autophagy, sepsis-induced acute lung injury, bioinformatics

## Abstract

Sepsis-induced acute lung injury is associated with lung epithelial cell injury. This study analyzed the role of the antimicrobial peptide LL37 with mitochondrial DNA (LL37–mtDNA) and its potential mechanism of action in lipopolysaccharide (LPS)-treated rat type II alveolar epithelial cells (RLE-6TN cells). RLE-6TN cells were treated with LPS alone or with LL37–mtDNA, followed by transcriptome sequencing. Differentially expressed and pivotal genes were screened using bioinformatics tools. The effects of LL37–mtDNA on cell viability, inflammation, apoptosis, reactive oxygen species (ROS) production, and autophagy-related hallmark expression were evaluated in LPS-treated RLE-6TN cells. Additionally, the effects of Hsp90aa1 silencing following LL37–mtDNA treatment were investigated in vitro. LL37–mtDNA further suppressed cell viability, augmented apoptosis, promoted the release of inflammatory cytokines, increased ROS production, and elevated LC3B expression in LPS-treated RLE-6TN cells. Using transcriptome sequencing and bioinformatics, ten candidate genes were identified, of which three core genes were verified to be upregulated in the LPS + LL37–mtDNA group. Additionally, Hsp90aa1 downregulation attenuated the effects of LL37–mtDNA on LPS-treated RLE-6TN cells. Hsp90aa1 silencing possibly acted as a crucial target to counteract the effects of LL37–mtDNA on viability, apoptosis, inflammation, and autophagy activation in LPS-treated RLE-6TN cells.

## 1 Introduction

Sepsis, a severe clinical infectious disease, is characterized by an uncontrolled systemic inflammatory response syndrome. Acute lung injury is considered a common fallout with a mortality rate of up to 30–40% that has greatly affected human health [1,2]. Owing to its complicated and unclear pathogenesis, lung-protective measures are currently deemed the main clinical therapies for sepsis-induced acute lung injury [2]. Alveolar epithelial cells serve as protective hubs for the regulation of lung function [3]. Nevertheless, excessive inflammation and damage to alveolar epithelial cells due to intra- or extra-pulmonary factors are regarded as the key pathogenic mechanisms of acute lung injury [4]. Therefore, it is necessary to elucidate the mechanisms underlying lung epithelial cell injury to identify novel perspectives for the treatment of sepsis-induced acute lung injury.

Autophagy is a homeostatic cellular process that removes unnecessary or damaged components, allowing their degradation and recycling into fundamental constituents for other cellular functions [5,6]. Increasing evidence indicates that dysregulation of autophagy is implicated in the etiology of several pulmonary diseases involving epithelial cells [7]. For instance, the activation of autophagy targeting alveolar epithelial cells contributes to protection against alveolar barrier dysfunction in acute lung injury mice [8]. RAGE depletion attenuates lipopolysaccharide (LPS)-induced lung injury by directly inhibiting autophagic apoptosis in type II alveolar epithelial cells [9]. In addition, astragaloside IV mitigates idiopathic pulmonary fibrosis by facilitating the initiation of autophagy in TGF-β1 treated alveolar epithelial cells [10]. Therefore, targeting autophagy may represent a novel strategy for ameliorating lung epithelial cell injury. Thus, further in-depth exploration is necessary.

The human antimicrobial peptide LL37, which belongs to the cathelicidin family, is synthesized by neutrophils and epithelial cells [11,12]. LL37 exerts antimicrobial properties and modulates inflammatory responses and the innate immune system [12]. Moreover, LL37 can form complexes with self-DNA, which plays a crucial role in the development of therapeutic strategies against HIV infection and atopic dermatitis [13,14]. Additionally, LL37 with endogenous mitochondrial DNA (mtDNA) (LL37–mtDNA) promotes atherosclerosis progression by enhancing autophagy recognition [15]. However, the specific functions of LL37–mtDNA and its regulatory effect on autophagy, as well as the molecular mechanisms by which LL37–mtDNA regulates sepsis-induced acute lung injury remain largely unclear.

Currently, the properties of LL37–mtDNA and its underlying mechanisms in sepsis-induced acute lung injury remain unclear. The novelty of this study lies in illuminating that LL37–mtDNA inhibited cell viability, augmented apoptosis, stimulated inflammatory cytokine release, and intensified autophagy in LPS-treated rat type II alveolar epithelial (RLE-6TN) cells, which were partially abolished by Hsp90aa1 knockdown.

## 2 Materials and methods

### 2.1 Cell culture, treatment, and transfection

RLE-6TN and HEK293 cells were procured from iCell (Shanghai, China). The cells were maintained in Dulbecco’s modified Eagle’s medium (DMEM; Hyclone, Logan, UT) containing 10% fetal bovine serum (Hyclone), 100 U/mL penicillin, and 100 μg/mL streptomycin (Hyclone) at 37°C with 5% CO_2_. RLE-6TN cells were stimulated with LPS (Sigma-Aldrich, Merck KGaA, Darmstadt, Germany) at a dose of 1 μg/mL for 6 h, followed by co-treatment with mtDNA (2 μg/mL) and antimicrobial peptide LL37 (10 μg/mL, AnaSpec, CA).

After reaching approximately 70–80% confluence, Hsp90aa1 siRNAs and negative control (si-NC) were transfected into RLE-6TN cells using Lipofectamine 3000 (Invitrogen, Carlsbad, CA). All the sequences were designed by GenePharma (Shanghai, China) and are listed in Table S1.

### 2.2 Preparation of mtDNA

mtDNA isolation kits (K280-50, BioVision) were used to extract and purify mtDNA from the cultivated HEK293 cells. The concentration of isolated mtDNA was determined using a spectrophotometer (Nanodrop ND-1000, Thermo Fisher Scientific, Waltham, MA, USA), and the obtained mtDNA was diluted for subsequent experiments.

### 2.3 Cell counting kit-8 (CCK-8) assay

RLE-6TN cells were seeded in 96-well microplates at a density of 1 × 10^4^ cells/well and cultivated in complete DMEM in an incubator at 37°C for 24 h. After treatment, the cells were treated with 10 μL of CCK-8 reagent (Beyotime, Shanghai, China) for 2 h. The absorbance was measured at 450 nm using a microplate reader (VL0000D0, Thermo Fisher Scientific).

### 2.4 Cell apoptosis

RLE-6TN cells were inoculated in six-well plates at a density of 5 × 10^5^/well at 37°C for 24 h. After different treatments, the cells were collected, centrifuged, and resuspended, and 5 μL AnnexinV-FITC/PI (Beyotime) was added for a 15 min incubation period in the dark. Finally, data were analyzed using flow cytometry (BD Biosciences, San Jose, CA).

### 2.5 Measurement of inflammatory cytokines and reactive oxygen species (ROS)

The culture medium from RLE-6TN cells with different treatments was harvested and centrifuged, and the obtained supernatant was transferred to new centrifuge tubes for subsequent detection. The production of inflammatory cytokines including interleukin-1β (IL-1β) and tumor necrosis factor-α (TNF-α) in the supernatant was measured using specific enzyme-linked immunosorbent assay kits (Esebio, Shanghai, China). Additionally, the ROS content in the supernatant was determined using the ROS assay kit (Solarbio, Beijing, China) and a microplate reader (Thermo Fisher Scientific).

### 2.6 Western blotting

Total protein samples from RLE-6TN cells of each group were extracted, harvested, quantified, passed through sodium dodecyl sulfate polyacrylamide gel electrophoresis, and transferred to polyvinylidene fluoride membranes. After blocking with 5% skimmed milk for 2 h, the membranes were incubated with specific primary antibodies against LC3B (ab192890, 1:2,000; Abcam, Cambridge, UK), Dhx9 (ab183731, 1:1,000; Abcam), Hsp90aa1 (4877, 1:1,000; Cell Signalling Technology, Danvers, MA), Sf3b1 (ab172634, 1:1,000; Abcam), and GAPDH (5174, 1:1,000; Cell Signalling Technology) at 4°C overnight. Following incubation with the corresponding secondary antibody, the fluorescence signals of the membranes were detected using an imager system (Bio-Rad, Hercules, CA, USA), with GAPDH serving as an endogenous control, and quantified using ImageJ software (V1.8.0.112, NIH, Madison, WI, USA).

### 2.7 Quantitative real-time polymerase chain reaction (qRT-PCR)

Total RNA was isolated from RLE-6TN cells of each group using TRIzol reagent (Invitrogen, Carlsbad, CA, USA), and the resulting RNA was reverse transcribed using the PrimeScript RT Reagent kit (TaKaRa, Otsu, Japan). The qRT-PCR analysis was conducted on the ABI7500 quantitative PCR instrument (Thermo Fisher Scientific) with the following cycling characteristics: 95°C for 30 s and 40 cycles of 95°C for 10 s and 60°C for 30 s. A comparative 2^−ΔΔCt^ method was applied to determine changes in the relative expression of different samples, with GAPDH as the housekeeping gene. The primer sequences used were designed by Shenggong Bioengineering Company (Shanghai, China) and are listed in Table S1.

### 2.8 Transcriptome sequencing

Transcriptome sequencing was performed by GeneDenovo Biotechnology Co., Ltd. (Guangzhou, China). Briefly, total RNA was extracted from RLE-6TN cells of each group (three each for the LPS and LPS + LL37–mtDNA groups) using TRIzol reagent (Invitrogen). The concentration and purity of RNA were determined using NanoDrop 2000 (Thermo Fisher Scientific) and RNA integrity was detected using an Agilent 2100 Bioanalyzer (Agilent Technologies, Santa Clara, CA, USA, RIN ≥ 7.0). Subsequently, libraries were constructed and sequenced using the Illumina NovaSeq 6000 platform (Illumina, San Diego, CA, USA). The clean reads were aligned with the reference genome using hisat2.

### 2.9 Bioinformatics analysis

The principal component analysis (PCA) was conducted to validate the reproducibility of the transcriptome sequencing sample data. The differentially expressed genes (DEGs) were filtered with adjusted *p* < 0.05 and |log_2_fold change| > 2 as the cut-off criteria. The “ggplot2” and “Pheatmap” packages in the R software were applied to visualize the DEGs. By analyzing the intersection of DEGs with mitophagy-related genes, a Venn diagram was generated using the EVenn tool (http://www.ehbio.com/test/venn/#/).

Gene Ontology (GO) annotation and Kyoto Encyclopedia of Genes and Genome (KEGG) analysis were conducted using “clusterProfiler” R package, and the results were depicted in the bubble plots with *p* < 0.05 considered significantly enriched.

The Search Tool for the Retrieval of Interacting Genes (STRING, https://www.string-db.org) was adopted to generate a protein–protein interaction (PPI) network with a confidence of 0.4 as the significance criterion. Afterward, the Molecular Complex Detection (MCODE) plugin of Cytoscape software was used for identifying key modules, and the focal genes were discovered using the degree algorithm of CytoHubba with the connectivity degree ≥10.

### 2.10 Statistical analysis

GraphPad Prism 9.0 (GraphPad Software Inc., San Diego, CA, USA) was used for statistical analysis with quantitative data presented as mean ± standard deviation. Student’s *t*-test was applied to assess differences between two sets of data, while the differences among multiple groups were compared using one-way analysis of variance in combination with Tukey’s test. Each experiment had at least three independent replicates, and significant differences were accepted when *p* <  0.05.

## 3 Results

### 3.1 Effects of LL37–mtDNA on LPS-treated RLE-6TN cells

**Figure 1:**
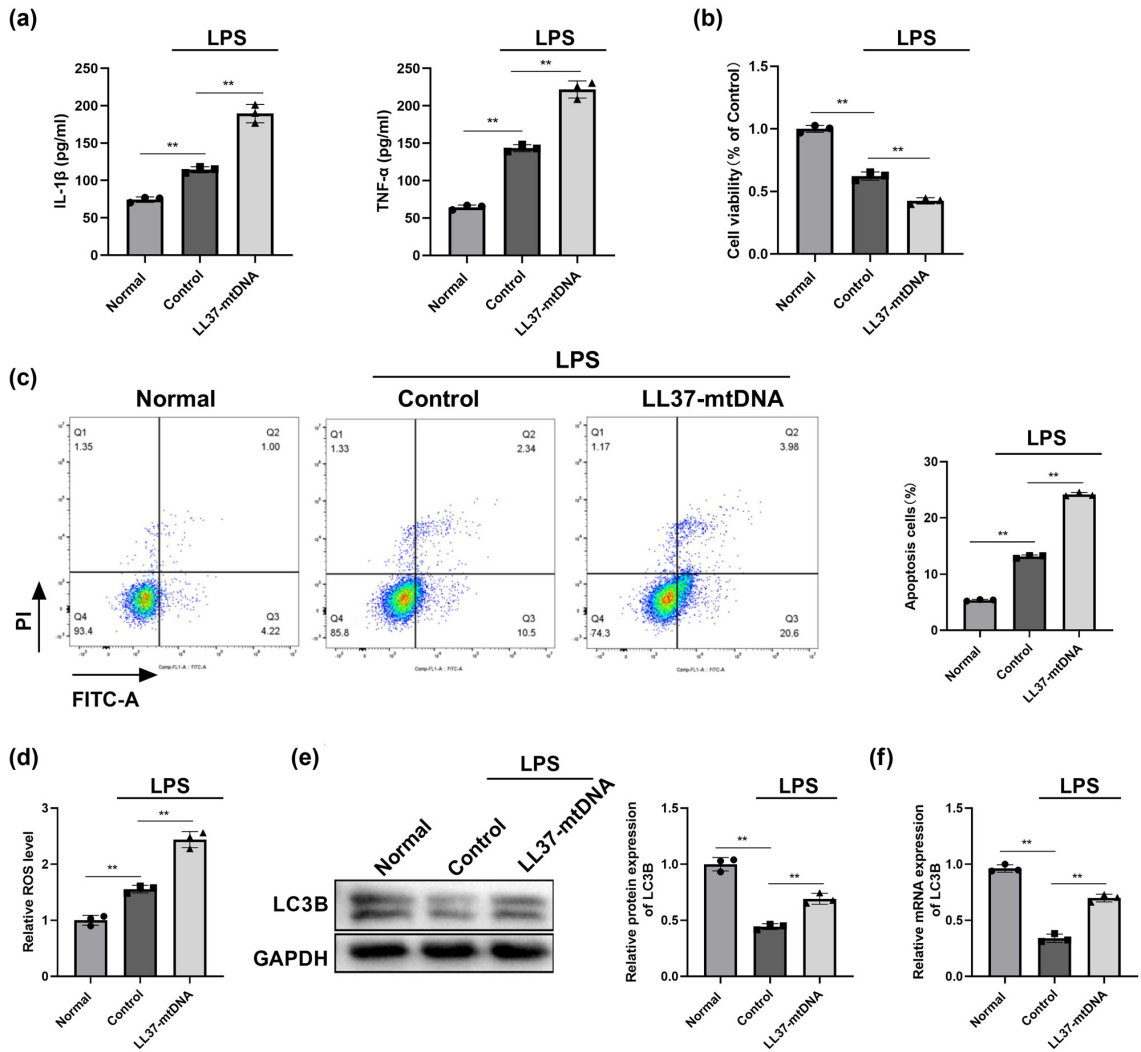
Effects of LL37–mtDNA in LPS-treated RLE-6TN cells. (a) The contents of Interleukin-1β (IL-1β) and tumor necrosis factor-α (TNF-α) of each group were detected using enzyme-linked immunosorbent assay (ELISA). (b) Cell viability was detected by cell counting kit-8 (CCK-8) assay. (c) Flow cytometry was performed to detect cell apoptosis. (d) The relative reactive oxygen species (ROS) level was determined with ROS assay kit. (e) The protein expression of LC3B was detected by western blot. (f) The relative mRNA expression of LC3B was detected by quantitative real-time polymerase chain reaction (qRT-PCR). **p ＜ 0.01.

We first determined the effects of LL37–mtDNA on LPS-treated RLE-6TN cells. LPS-induced release of IL-1β and TNF-α was aggravated by LL37–mtDNA treatment in RLE-6TN cells (Figure 1a). The viability of LPS-treated RLE-6TN cells decreased, and LL37–mtDNA treatment further suppressed this effect (Figure 1b). Subsequently, we found that the apoptotic cell rate increased in LPS-treated RLE-6TN cells and was more pronounced in the LL37–mtDNA + LPS group (Figure 1c). In addition, the ROS level and the protein and mRNA expression levels of LC3B were downregulated in LPS-treated RLE-6TN cells, whereas this trend was blocked by LL37–mtDNA treatment (Figure 1d–f), implying that LL37–mtDNA inhibited viability and promoted apoptosis, inflammation, and autophagy in LPS-treated RLE-6TN cells.

### 3.2 Identification of DEGs and functional enrichment analysis

**Figure 2:**
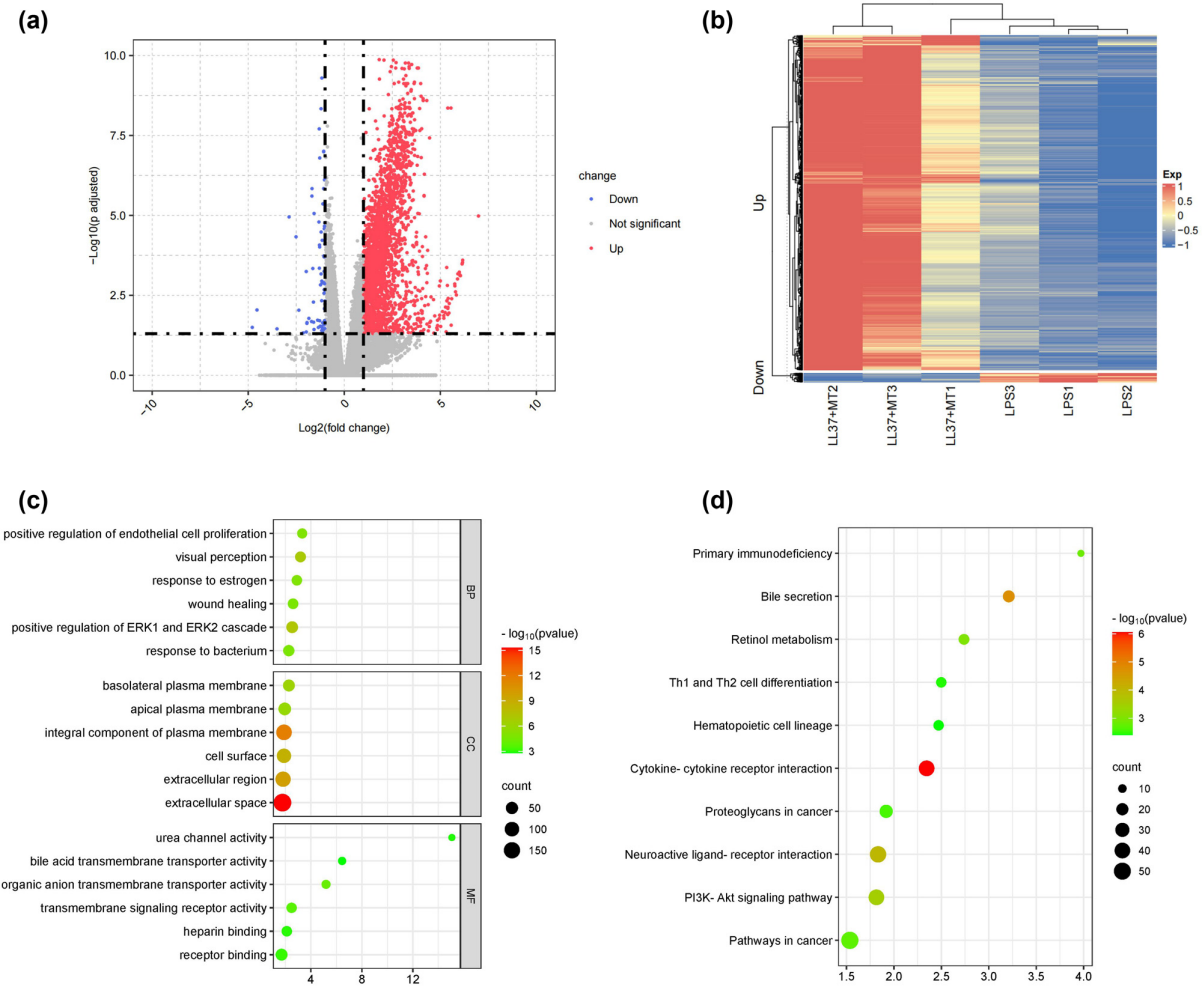
Identification of differentially expressed genes (DEGs) and functional enrichment analysis. (a) Volcano plot highlighting DEGs (3 each for the LPS and LPS + LL37–mtDNA groups). Red, blue, and gray nodes separately indicate up-regulated, down-regulated and none significantly changed genes. (b) The heat maps of DEGs between LPS and LPS + LL37–mtDNA group. The upregulated DEGs are displayed in red, and the downregulated in blue. (c) Gene Ontology (GO) functional analysis bubble plot. Top 6 GO enrichment terms in biological process (BP), cellular component (CC), and molecular function (MF) categories of the DEGs, respectively. (d) Top ten enriched pathways of DEGs in the Kyoto Encyclopedia of Genes and Genome (KEGG) database.

The PCA showed that the reproducibility of the transcriptome sequencing sample data was modestly good (Figure S1). There were 1721 DEGs between the LPS and LPS + LL37–mtDNA treatment groups, including 1694 upregulated and 27 downregulated DEGs (Figure 2a). The heatmap shows the gene expression levels of significant DEGs in LPS- and LPS + LL37–mtDNA-treated samples (Figure 2b). The top 15 upregulated and downregulated DEGs are provided in Table S2.

To further investigate the potential functional roles of DEGs, GO and KEGG analyses were conducted. The top enriched six GO terms with the lowest *p*-values are shown for each item of biological process, including visual perception, positive regulation of endothelial cell proliferation, cell composition such as extracellular space and integral component of plasma membrane, and molecular function such as urea channel activity. (Figure 2c, Table S3). For KEGG analysis, the top ten pathways with the lowest *p*-values are presented, such as the PI3K–Akt signaling pathway (Figure 2c, Table S4).

### 3.3 Construction of PPI network and hub gene selection

**Figure 3:**
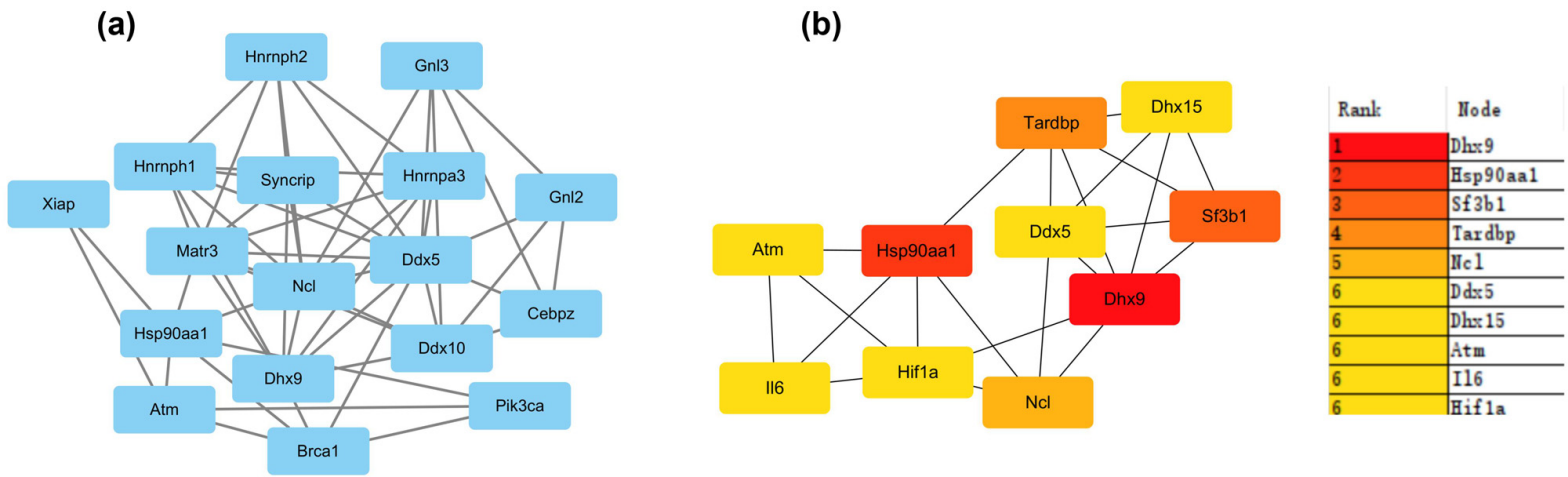
Identification of the hub genes. (a) One key cluster was identified by MCODE. (b) Top ten hub genes were determined by degree algorithm using CytoHubba. The deeper the red node, the higher the degree of connectivity.

A Venn diagram was generated between DEGs and mitophagy-associated genes, and 250 mitophagy-related DEGs were screened (Figure S2a), followed by PPI network construction using the STRING network analysis tool (Figure S2b). Subsequently, one tightly connected cluster module was obtained using the MCODE plugin (Figure 3a), and the top ten ranked hub genes were extracted using the degree algorithm in the CytoHubba plugin (Figure 3b).

### 3.4 Quantification of key hub genes

**Figure 4:**
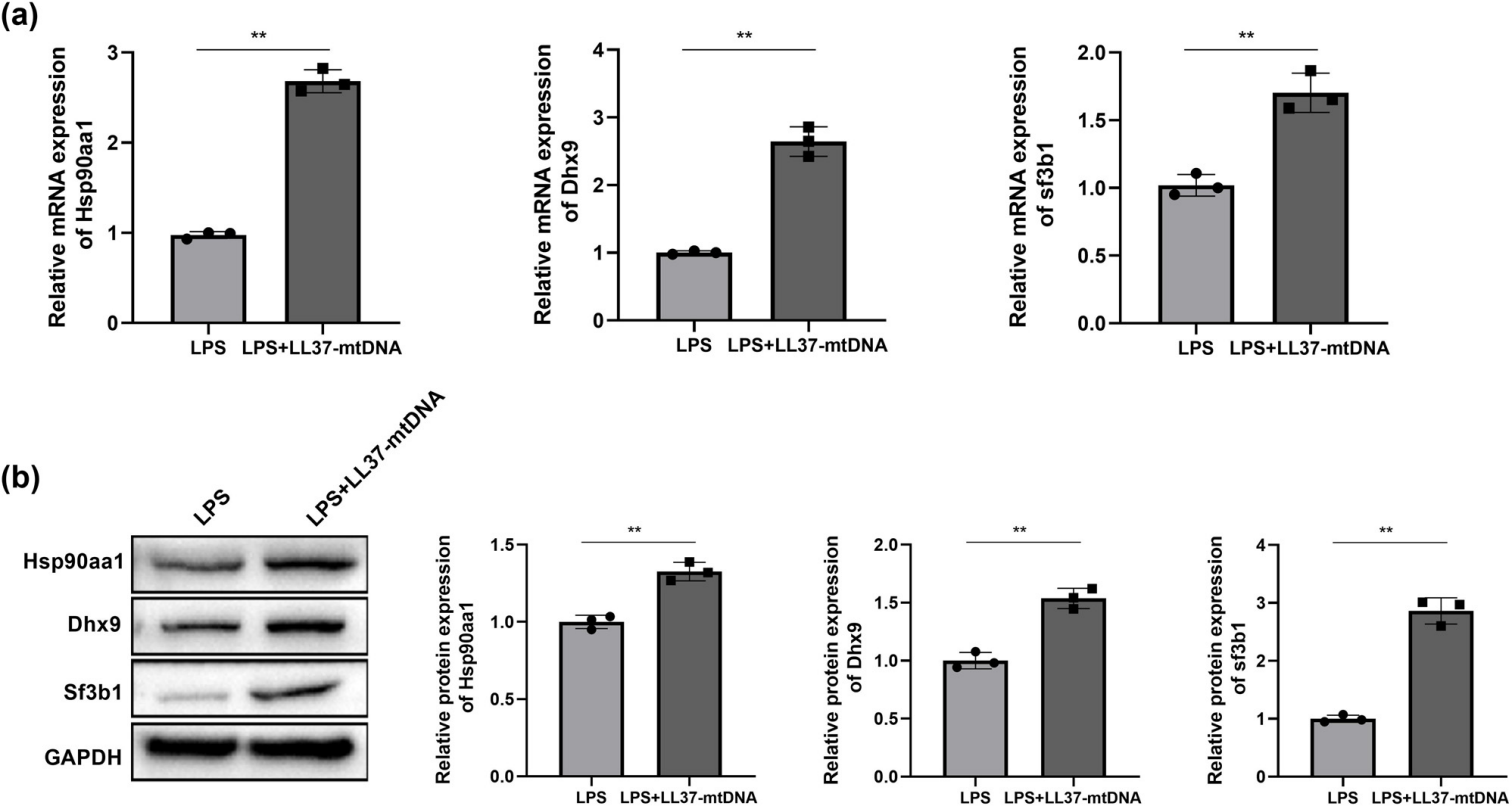
Quantification of hub genes. (a) QRT-PCR was applied to evaluate the expression of Hsp90aa1, Dhx9, and Sf3b1 at mRNA levels. (b) Protein levels of Hsp90aa1, Dhx9, and Sf3b1 were verified by western blot. **p ＜ 0.01 vs LPS.

To verify our bioinformatics analysis results, we validated the expression levels of three hub genes in RLE-6TN cells treated with LPS alone or in combination with LL37–mtDNA. Compared with LPS treatment alone, the relative mRNA expression of Hsp90aa1, Dhx9, and Sf3b1 was remarkably upregulated in the LPS + LL37–mtDNA group (Figure 4a). Consistently, the protein expression levels of these three genes were elevated in the LPS + LL37–mtDNA group (Figure 4b).

### 3.5 LL37–mtDNA facilitates inflammation, inhibits viability, promotes apoptosis, and enhances autophagy by targeting Hsp90aa1 in LPS-treated RLE-6TN cells

**Figure 5:**
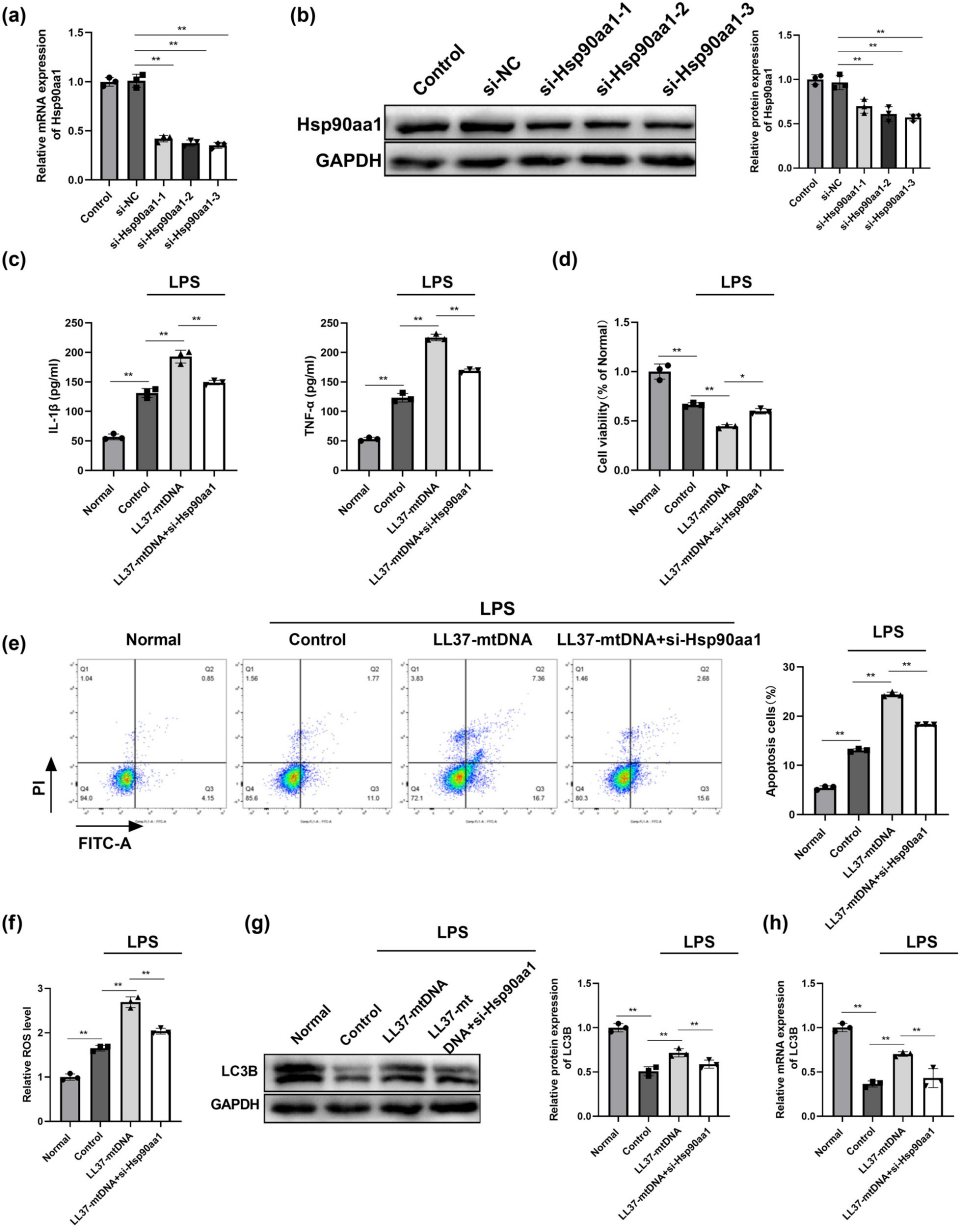
LL37–mtDNA restrains viability, promotes apoptosis and inflammation, and enhances autophagy by targeting Hsp90aa1 in LPS-treated RLE-6TN cells. (a) QRT-PCR assay was utilized to verify the knockdown efficiency of Hsp90aa1 expression. The expression of Hsp90aa1 was downregulated in the RLE-6TN cells compared to the si-NC. (b) Western blot was applied to measure the knockdown efficiency of Hsp90aa1 expression. The expression of Hsp90aa1 was downregulated in the RLE-6TN cells compared to the si-NC. (c) The levels of IL-1β, TNF-α, and ROS in the supernatant of each group were determined. (d) Viability of RLE-6TN cells after different treatments using the CCK8 assay. (e) Cell apoptosis of RLE-6TN cells in each group was analyzed by flow cytometry. (f) The ROS level in each group was examined using matched assay kits. (g) The protein level of LC3B in each group was evaluated using the Western blot. (h) The mRNA level of LC3B in each group was evaluated using qRT-PCR. **p < 0.01.

Next, we investigated the potential capabilities of Hsp90aa1. Hsp90aa1 was silenced in RLE-6TN cells, and the expression of Hsp90aa1 after transfection with si-Hsp90aa1-3 was the lowest compared with that in si-NC; therefore, si-Hsp90aa1-3 was selected for further experiments (Figure 5a and b). The LL37–mtDNA + si-Hsp90aa1 group exhibited lower levels of IL-1β, and TNF-α than those in the LL37–mtDNA group (Figure 5c). Hsp90aa1 silencing attenuated the inhibitory effect of LL37–mtDNA treatment on the viability of LPS-treated RLE-6TN cells (Figure 5d). The pro-apoptotic effect of LL37–mtDNA treatment was partially reversed by Hsp90aa1 knockdown in LPS-treated RLE-6TN cells (Figure 5e). Additionally, LL37–mtDNA treatment increased ROS level and LC3B expression, which was suppressed by Hsp90aa1 downregulation in LPS-treated RLE-6TN cells (Figure 5f–h). Collectively, LL37–mtDNA facilitated inflammation, restrained viability, promoted apoptosis, and enhanced autophagy by targeting Hsp90aa1 in LPS-treated RLE-6TN cells.

## 4 Discussion

Sepsis-induced acute lung injury may be attributed to the dysregulation of alveolar type II cells [16,17]. Hence, further investigation of the molecular mechanisms involved in pulmonary epithelial cell injury is of immense importance, and it may be leveraged to alleviate the development of sepsis-induced acute lung injury. In this study, combining transcriptome sequencing technology and comprehensive bioinformatics methods, we screened the top ten candidate genes between LPS alone and LPS + LL37–mtDNA treated samples. Furthermore, our results revealed that LL37–mtDNA inhibited cell viability, augmented apoptosis, facilitated inflammation, and enhanced autophagy in LPS-treated RLE-6TN cells, whereas these phenomena were partially abolished after Hsp90aa1 knockdown.

LL37 exerts a protective role against lung injury in LPS + ATP-treated alveolar epithelial cells by ameliorating the severe inflammatory response, as reflected by reduced expression levels of IL-1β and IL-18 [18]. In addition, the LL37–bacDNA complex facilitates inflammatory cytokine secretion, thus aggravating the severity of ulcerative colitis [19]. Moreover, the LL37–DNA complex greatly increases the production of IFN-α in cultured keratinocytes, indicating a link between LL37–DNA and autoinflammation in cholesteatoma [20]. In addition, LL37–mtDNA complex can deteriorate lung inflammation in septic mice with acute lung injury by impairing autophagy recognition [21]. Here, we found that LL37–mtDNA suppressed cell viability, augmented apoptosis, and promoted the production of inflammatory factors in LPS-treated RLE-6TN cells, indicating that LL37–mtDNA exacerbates alveolar epithelial cell injury.

As suggested by our KEGG analysis, the Pl3K–Akt signaling pathway was primarily enriched pathways that participated in LL37–mtDNA-treated cells. Tian et al. have found that the PI3K/Akt/HO-1 pathway is involved in the mediation of autophagy during sepsis-induced lung injury in mice [22]. Nonetheless, herein, major pathways in which overlapping DEGs are abundant were not related to apoptosis, autophagy, or ROS production. Bioinformatics tool is a viable option to predict possible pathways, which may help better understand the development of lung epithelial cell injury. However, possibly due to the small sample size and differences in transcriptome sequencing data quality, the results were easily affected, resulting in bias, we could not verify the obtained conclusions through bioinformatics; thus, more samples are necessary to further confirm the impact of LL37–mtDNA. A PPI network was constructed and three core genes, *Dhx9*, *Hsp90aa1*, and *Sf3b1*, were confirmed. Therefore, long noncoding RNA SH3PXD2A-AS1 interacts with *Dhx9* to facilitate proliferation and cell cycle progression in non-small-cell lung carcinoma [23]. Moreover, *Dhx9* is prominently linked to the prognosis of patients with lung tumors [24]. Additionally, *Sf3b1* is involved in the prognosis of non-small-cell lung carcinoma [25]. In the present study, *Dhx9* and *Sf3b1* expression was upregulated in LPS + LL37–mtDNA-treated RLE-6TN cells, and the detailed mechanisms by which LL37–mtDNA regulates lung epithelial cell injury require further elucidation.

Notably, Hsp90aa1 is an isoform of the molecular chaperone Hsp90 which significantly regulates signal transduction, proliferation, cell cycle, apoptosis, and tumor progression [26,27]. Hsp90aa1 is downregulated in the blood and human osteoarthritic cartilage and is negatively associated with the risk of osteoarthritis [28]. Conversely, Hsp90aa1 is overexpressed in patients with lung cancer, and downregulation of Hsp90aa1 promotes cell apoptosis and inhibits proliferative ability by inhibiting the AKT1/ERK pathway [29]. Additionally, Hsp90aa1 was significantly elevated in the lung tissues of an idiopathic pulmonary fibrosis rat model and is considered a novel system biomarker [30]. Consistently, we demonstrated that the expression of Hsp90aa1 was increased in RLE-6TN cells co-treated with LPS and LL37–mtDNA. In addition, downregulation of Hsp90aa1 ameliorated the effects of LL37–mtDNA treatment on viability, apoptosis, and inflammatory cytokine release in LPS-treated RLE-6TN cells, suggesting that Hsp90aa1 silencing may be an attractive therapeutic target for lung epithelial cell injury.

The exact role of autophagy in the pathophysiology of LPS-induced acute lung injury remains somewhat controversial. Autophagy possibly exerts protective effects in LPS-induced inflammatory lung injury [31]. lncRNA-SNHG14 is participated in alveolar type II epithelial cell injury induced by LPS through controlling the occurrence of autophagy [32]. Ketamine facilitates autophagy in alveolar type II epithelial cells after LPS treatment and in a sepsis-induced acute lung injury mouse model by regulating AMPK/mTOR pathway activation [33]. Isorhamnetin can also protect type II alveolar epithelial cells against LPS-induced injury by activating autophagy via the inhibition of the mTOR pathway [34]. Inositol enhances autophagy activation by ameliorating LPS-treated alveolar epithelial cell inflammation, which is attributed to the regulation of the HIF-1 α-SLUG axis [35]. Conversely, long-term or ineffective autophagy may harm the lung epithelial cells and exacerbate lung damage [36]. Through the suppression of the PI3K/Akt/mTOR pathway, aspirin activates autophagy, thereby promoting pulmonary fibrosis [37]. In the present study, we found that LL37–mtDNA treatment contributed to enhanced autophagy activation, as reflected by the partially reversal of LC3B reduction triggered by LPS in RLE-6TN cells after using LL37–mtDNA. Nonetheless, these phenomena were further suppressed by Hsp90aa1 downregulation. These data imply that LL37–mtDNA may enhance autophagy by modulating Hsp90aa1 expression in a sepsis-induced acute lung injury cell model.

However, this study had some limitations. We preliminarily explored the effects of LL37–mtDNA on lung epithelial cell injury but investigated only one lung epithelial cell line. Furthermore, the impact of LL37–mtDNA lacked animal experiments and clinical verification. In addition, more specific molecular mechanisms and interactions with LL37–mtDNA should be elucidated.

In summary, ten hub genes were extracted by combining transcriptome sequencing technology and comprehensive bioinformatics analysis, among which three core genes were confirmed to be upregulated after co-treatment with LPS and LL37–mtDNA. Our data illustrated that LL37–mtDNA reduced cell viability, augmented apoptosis, stimulated inflammatory cytokine release, and intensified autophagy in LPS-treated RLE-6TN cells, while these changes were partially counteracted by Hsp90aa1 silencing. Overall, the findings of this study suggested that Hsp90aa1 knockdown is a potential novel candidate target for ameliorating lung epithelial cell injury, which may further novel insights into the etiopathogenesis of sepsis-induced acute lung injury.

## Supplementary Material

Supplementary material
