# Supplementary material for: LL37-mtDNA regulates viability, apoptosis, inflammation, and autophagy in lipopolysaccharide-treated RLE-6TN cells by targeting Hsp90aa1
Source: Open Life Sci. 2024 Aug 28;19(1):20220943. doi: 10.1515/biol-2022-0943 (PMC11365468; doi:10.1515/biol-2022-0943)
Supplement: Supplementary material [file biol-2022-0943-sm.pdf]

Supplementary material

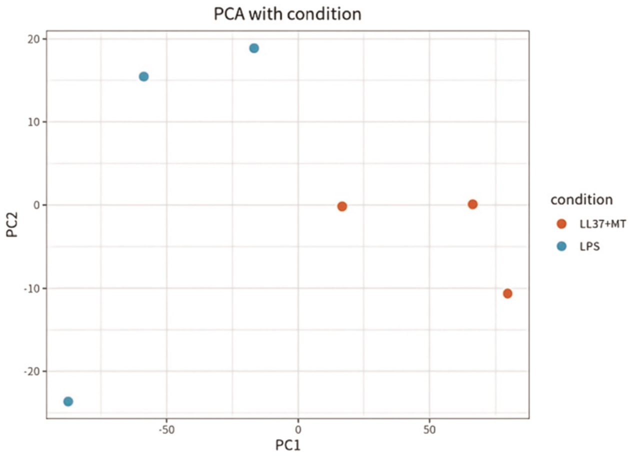

Figure S1: Principal component analysis of the data (three each for the LPS and LPS + LL37-mtDNA groups).

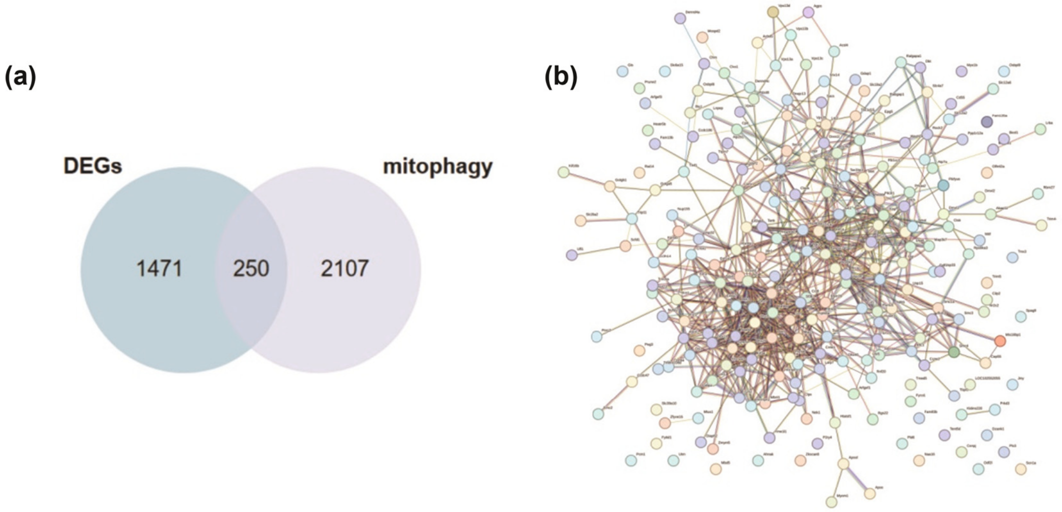

Figure S2: Construction of protein-protein interaction (PPI) network based on DEGs related to mitophagy. (a) Venn diagram of DEGs related to mitophagy. (b) The PPI network was generated by the Search Tool for the Retrieval of Interacting Genes (STRING) database.

Table S1: The sequences of primers and siRNAs used for quantitative real-time polymerase chain reaction (qRT-PCR)

Name	Sequences（5′-3′）
GAPDH-F	GAGTCAACGGATTTGGTCGT
GAPDH-R	TTGATTTTGGAGGGATCTCG
LC3B-F	TGTCCAGGGGTAACTTGGTC
LC3B-R	AGCTGTCAGAAACGCCCTTA
Hsp90aa1-F	AGGTCGAAACCTTTGCCTTT
Hsp90aa1-R	TTCCAATGCCAGTATCCACA
Dhx9-F	ATGGGCAATTCCACCAATAA
Dhx9-R	GCTTTGAGAGCCAGATGAGG
Sf3b1-F	GATGAGACCCCCAAAACAGA
Sf3b1-R	AGTGCTTCCACCCATTTGAC
si-NC-F	UUCUCCGAACGUGUCACGUTT
si-NC-R	ACGUGACACGUUCGGAGAATT
si-Hsp90aa1-1-F	AGAAAGAUCUCUUUGUUCGAG
si-Hsp90aa1-1-R	CGAACAAAGAGAUCUUUCUGA
si-Hsp90aa1-2-F	UCAAACAGAUCAAAAGGAGCG
si-Hsp90aa1-2-R	CUCCUUUUGAUCUGUUUGAAA
si-Hsp90aa1-3-F	UUUUCAAACAGAUCAAAAGGA
si-Hsp90aa1-3-R	CUUUUGAUCUGUUUGAAAACA

Table S2: Top 15 up-regulated and down-regulated differentially expressed genes (DEGs) in LPS and LPS+LL37-mtDNA treatment samples

Name	log2FoldChange	pval	up/down
Serpinc1	6.985805842	4.61491×10^{-7}	up
Flvcr2	6.029044288	9.15243×10^{-5}	up
Ccdc141	6.025279961	9.57427×10^{-5}	up
AABR07063703.1	5.988681261	6.92049×10^{-5}	up
Mob1a-ps1	5.759031376	0.000251763	up
Evi2b	5.465491125	0.001333735	up
H2az1-ps2	5.450841547	0.003255027	up
Cyld-ps1	5.398566834	0.001901736	up
Tent5d	5.392025678	0.002114315	up
Chm	5.39022252	1.98388×10^{-11}	up
Nufip2-ps1	5.387478767	0.003758715	up
Fancd2os	5.342723829	0.001033281	up
Tnfsf15	5.2749315	0.001871375	up
AABR07004881.1	5.18368658	0.005297903	up
AABR07008066.2	5.180846826	0.004183245	up
Cd300lf	−4.788375238	0.012347781	down
Mafa	−4.543524243	0.002466006	down
Gdf5	−4.112050238	0.046414754	down
Ntrk3	−4.082735236	0.048066516	down
Kcnk16	−4.082735236	0.048066516	down
Calb2	−3.465269281	0.022991884	down
Rpl21	−2.789490383	0.022813877	down
Aspdh	−2.518960816	0.030061284	down
Cfd	−2.504048542	0.033803012	down
RGD1560986	−2.486541355	0.036384667	down
Garin5a	−2.141658481	0.020226546	down
Atp5mk-ps2	−1.978547602	6.5397×10^{-5}	down
Trim47	−1.976880391	0.018482647	down
Olfml2a	−1.939508063	0.007908425	down
Cilp2	−1.841804739	0.005363038	down

Table S3: Gene Ontology (GO) TERM for DEGs between the LPS and LPS+LL37-mtDNA groups

GOTERM	Term	*P* Value	Genes
GOTERM_BP_DIRECT	positive regulation of ERK1 and ERK2 cascade	6.32×10^{-8}	NPSR1, CSF1R, EPO, HTR2B, PDGFB, CXCR4, PTPN22, FGF2, C1QTNF3, CCL9, GPNMB, CCL5, KDR, CCL2, ALKAL2, CCL1,
GOTERM_BP_DIRECT	visual perception	1.34×10^{-7}	SLC24A1, LAMC3, CABP4, CRYBA1, NR2E1, PDC, RS1, GABRR2, ZIC2, RDH12, OPA3, RHO, PDE6A, IMPG2, GPR179
GOTERM_BP_DIRECT	response to estrogen	1.07×10^{-5}	TPH2, ABCC2, SLC10A1, EPO, CAV1, IGFBP2, PRKAG1, PDGFB, WNT7A, OPRK1, TNFRSF11B,
GOTERM_BP_DIRECT	positive regulation of endothelial cell proliferation	1.15×10^{-5}	PPP1R16B, ACVRL1, SEMA5A, JUN, CAV1, HTR2B, PDGFB, PIK3CD, VEGFD, CYBA, PROX1,
GOTERM_BP_DIRECT	response to bacterium	1.53×10^{-5}	MIR30B, CD40, NCF1, SLFN4, RNU1-123, NEXN, REG3G, MIR30C1, IKZF3, MIR126A, UPK1B, RGS1, CCL5, GPC3, CCL2, SCN7A, MIR192, PGLYRP1,
GOTERM_BP_DIRECT	wound healing	1.83×10^{-5}	SPARC, REG1A, PDGFB, F13A1, LTBP1, FGF2, GLI3, AQP1, FCGR3A, FGF7, MYOZ1, JAG1,
GOTERM_CC_DIRECT	extracellular space	4.80×10^{-16}	MIR30B, SPARC, SPINT3, C4BPA, RS1, LIPC, IL36B, PGLYRP1, RPL39, CHIA, WFDC21, RBP4, SLPI, PAPPA, PLBD1, RT1-S2,
GOTERM_CC_DIRECT	integral component of plasma membrane	1.75×10^{-12}	NPFFR2, CHRM4, AQP9, AQP7, SLC4A1, AQP2, CLDN1, SLCO2B1, MPZ, KDR, CMKLR2, SLC38A1, CASR, CHRNB3, DSCAM, EPHA8,
GOTERM_CC_DIRECT	extracellular region	1.46×10^{-10}	NRROS, F13A1, DEFB22, FGF2, RS1, SCGN, FGF7, ENPP2, PROK2, PGLYRP1, CAR6, APOL11A, HIST1H2AO, CHIA, FAM24B, IGFBP2, DIO2, HSPG2, PGF,
GOTERM_CC_DIRECT	cell surface	2.31×10^{-9}	ACVRL1, PLVAP, CIITA, CD40, NRROS, SLC46A2, SPARC, EPO, FLT3, CTSZ, MPL, FASLG,
GOTERM_CC_DIRECT	basolateral plasma membrane	6.02×10^{-7}	AABR07028997.1, AQP8, SLC22A2, AQP9, PDGFB, CLCNKA, AQP7, SLC4A1, SLC5A1, PTH1R, ATP12A,
GOTERM_CC_DIRECT	apical plasma membrane	1.59×10^{-6}	AQP8, PTPRO, PTH1R, ATP12A, AQP2, CLDN1, SLC4A5, SLC2A7, AQP1, UPK1B, SLCO2B1, PRKG2, SLC34A3, CASR, AMN, ANXA1,
GOTERM_MF_DIRECT	organic anion transmembrane transporter activity	7.20×10^{-5}	SLC22A25, ABCC2, SLCO1A3, SLCO1A4, SLCO1A1, SLCO2B1, SLC22A2, SLCO4C1, SLC22A8, SLC17A3
GOTERM_MF_DIRECT	transmembrane signaling receptor activity	1.77×10^{-4}	FCRL2, SPNL1, CHRNB3, NFAM1, GLRA4, SELE, CD3D, MRGPRB2, GABRR3, FCAMR, EDAR, CD79A, FCRLB, FCGR3A, SMO, PECAM1,
GOTERM_MF_DIRECT	receptor binding	6.97×10^{-4}	NXPH2, FGL1, REG1A, PDGFB, NTN5, LAMC2, ABCA12, DPP4, LBP, RSPO1, SKINT10, BTNL8,
GOTERM_MF_DIRECT	heparin binding	9.50×10^{-4}	AOC1, ECM2, SERPIND1, COL25A1, LAMC2, PRSS57, FGF2, FSTL1, COLQ, BMP4, FGF7, CCDC80, LIPC, GPNMB, CFHR2,
GOTERM_MF_DIRECT	urea channel activity	0.001108085	SLC14A1, AQP8, AQP9, AQP7
GOTERM_MF_DIRECT	bile acid transmembrane transporter activity	0.001540066	SLCO1A3, SLCO1A4, SLC10A1, SLCO1A1, SLCO2B1, SLC51B

Table S4: Kyoto Encyclopedia of Genes and Genome (KEGG) TERM for DEGs between the LPS and LPS+LL37-mtDNA groups

KEGG_PATHWAY	Term	*P* Value	Genes
KEGG_PATHWAY	Cytokine-cytokine receptor interaction	8.66×10^{-7}	ACVRL1, CSF1R, CXCL6, CSF3, CD40, EPO, MPL, CXCR4, FASLG, TNFRSF11B, CXCR6, TNFRSF13C, TNFSF13B,
KEGG_PATHWAY	Bile secretion	1.81×10^{-5}	ABCC2, UGT2B35, SLC10A1, AQP8, AQP9, SLC5A1, SLC51B, BAAT, ACNAT1, SLC4A5, AQP1,
KEGG_PATHWAY	Neuroactive ligand-receptor interaction	1.02×10^{-4}	NPSR1, NPFFR2, PTGER4, UCN, TAAR7E, CHRM4, TAAR7H, HTR2B, LPAR2, PTH1R, LPAR4, GABRR3, GRM3, GRM2, GABRR2,
KEGG_PATHWAY	PI3K-Akt signaling pathway	3.25×10^{-4}	CSF1R, CSF3, EPO, FLT3, LAMC3, PDGFB, PIK3CD, LPAR2, LAMC2, FASLG, LPAR4, FGF2, AREG, FGF7, CCND2, CCND1, KDR,
KEGG_PATHWAY	Retinol metabolism	9.75×10^{-4}	UGT2B35, HSD17B6, DHRS3, ADH6, SDR16C5, CYP26A1, CYP3A9, CYP2W1, RDH12, CYP2C11,
KEGG_PATHWAY	Primary immunodeficiency	0.001460649	ZAP70, CD79A, CIITA, CD40, CD8B, BTK, TNFRSF13C, IL7R, CD3D
KEGG_PATHWAY	Pathways in cancer	0.001918824	SPI1, WNT2B, EPO, LAMC3, FLT3, PIK3CD, FASLG, LAMC2, GLI1, FGF2, GLI3, FGF7, CCND2, CCND1
KEGG_PATHWAY	Proteoglycans in cancer	0.002624176	WNT2B, HPSE2, WNT8A, PIK3CD, FASLG, FGF2, CCND1, GPC3, KDR, WNT10B, WNT5B, ITGA2,
KEGG_PATHWAY	Th1 and Th2 cell differentiation	0.003548407	JUN, JAG1, RBPJL2, RT1-DOA, RT1-DOB, CD3D, MAPK13, ZAP70, MAF, IL2RA, TBX21, IL2RB
KEGG_PATHWAY	Hematopoietic cell lineage	0.003916278	CSF1R, CSF3, EPO, FLT3, ITGA2, RT1-DOA, RT1-DOB, CD3D, GP9, IL1A, CD8B, IL2RA, IL7R, CD44
